# Prediction of perturbed proton transfer networks

**DOI:** 10.1371/journal.pone.0207718

**Published:** 2018-12-12

**Authors:** Marco Reidelbach, Marcus Weber, Petra Imhof

**Affiliations:** 1 Institute for Theoretical Physics, Freie Universität Berlin, Berlin, Germany; 2 Mathematics for Life and Materials Sciences, Zuse Institue Berlin, Berlin, Germany; Hong Kong University of Science and Technology, HONG KONG

## Abstract

The transfer of protons through proton translocating channels is a complex process, for which direct samplings of different protonation states and side chain conformations in a transition network calculation provide an efficient, bias-free description. In principle, a new transition network calculation is required for every unsampled change in the system of interest, e.g. an unsampled protonation state change, which is associated with significant computational costs. Transition networks void of or including an unsampled change are termed unperturbed or perturbed, respectively. Here, we present a prediction method, which is based on an extensive coarse-graining of the underlying transition networks to speed up the calculations. It uses the minimum spanning tree and a corresponding sensitivity analysis of an unperturbed transition network as initial guess and refinement parameter for the determination of an unknown, perturbed transition network. Thereby, the minimum spanning tree defines a sub-network connecting all nodes without cycles and minimal edge weight sum, while the sensitivity analysis analyzes the stability of the minimum spanning tree towards individual edge weight reductions. Using the prediction method, we are able to reduce the calculation costs in a model system by up to 80%, while important network properties are maintained in most predictions.

## Introduction

The translocation of protons from one side of a biological membrane, e.g. the inner mitochondrial membrane, to the other is an exceptionally important process in nature [1]. To a small extent, protons are able to permeate through membranes on their own [2, 3]. The bulk of such translocations, however, occurs via proton-permeable channels [4]. One of the simplest channels is provided by *gramicidine* [5], a cation-selective, water-filled pore, which allows the translocation of protons at high rates [6]. In contrast to the translocation of other cations no translational motion of the water molecules within the channel is required [7], instead the proton translocation is believed to occur grotthus-like [8], i.e. proton “hops” between neighboring water molecules by exchanging covalent and hydrogen bonds to the donor and acceptor oxygen, along a single-file water chain [5]. Hence, a pore and a water chain (or a network of water molecules) is enough to facilitate the proton transfer. Interestingly, however, in another type of water-filled channels, the *aquaporines* [9], no proton permeation was observed [10]. Molecular dynamics (MD) simulations provided an explanation for these observations, revealing an interruption of the required water chain at an arginine, whose positive charge is repelling protons and thus preventing their translocation [11]. A more sophisticated regulation of the proton translocation was proposed for the *viral proton channel M2* [12]. Here, the side chains of four unprotonated histidine residues occlude the channel [13]. However, the protonation of at least two of them results in a conformational change, which allows the formation of a continuous water chain [13] and thus proton transfer. Such gates were also proposed for other proton transfer channels, e.g. the D-channel of *Cytochrome c Oxidase* (*CcO*) [14]. The proposed D-channel gate, however, is formed by an asparagine, rendering conformational changes due to a direct protonation hardly probable. Instead, MD simulations revealed a correlation between the conformation of the gate and the protonation state of other residues inside the channel [15, 16]. Furthermore, MD simulations revealed a correlation between the channel hydration and the protonation state of individual residues [17, 18]. Hence, protons seem to be able to alter important channel properties, while they are being transferred. To receive a comprehensive picture of the proton translocation through individual proton transfer channels, the well orchestrated interplay of local effects, e.g. conformational changes upon protonation, single proton “hops” with subsequent water shell re-orientations or proton induced water chain formations [18–21], and distant effects, e.g. conformational or hydrational changes upon protonation of distant residues, proton translocations along water chains, needs to be elucidated.

Due to the rareness of transition events, straight forward molecular simulations are not able to sample transition paths efficiently. Steered molecular simulations are only efficient and applicable, if it is possible to pre-define the relevant reaction coordinates of the described complex mechanisms. If it is not possible to anticipate molecular mechanisms, then path optimization methods are applicable [22]. Instead of trying to find the minimum energy path (MEP) by sampling, one can solve a corresponding optimization problem. On the basis of some initial guess-path a local optimization method determines the MEP in a very efficient way.

Over time a broad range of path optimization methods has been developed, e.g. the Nudged Elastic Band method (NEB) [23] with several modifications [24–26], the Conjugate Peak Refinement method (CPR) [27], the Ridge method [28], the DHS method [29], and the Dimer method (transition with unknown final state) [30]. While all these methods provide an estimate of the transition state, the NEB and CPR method provide a more global view on the energy surface [31]. Both methods allowed the determination of complex re-arrangements in proteins [32–35] as well as proton transfer events [36–38]. Another promising method is the string method [39], in which intrinsically parametrized curves evolve to the most probable transition pathway by following their dynamics, e.g. applied to the *RNA* backbone cleavage by *ribonuclease H* in which proton transfer reactions play a key role [40]. All these methods, however, tend to fail to represent the multitude of co-existing transition mechanisms [22], providing only the MEP whose transition states are closest to the initial guess-path [31]. To achieve a comprehensive description of the transition process nonetheless the state space can be partitioned into different substates, thus translating a complex reaction into a network of simpler transitions [22]. This Transition Network (TN) approach was successfully used to study small atom clusters or glasses [41, 42], peptides [43, 44] and complex protein transitions [45, 46]. Furthermore, we showed recently that TNs are also suitable to identify different proton transfer mechanisms, i.e. concerted/stepwise proton transfer, in a channel-like proton transfer system [47].

The TN approach characterizes the dynamical behavior of molecular systems by transition barriers, and thus, following the transition state theory [48], by transition rates. Other ways to express the same information are transition probabilities, like it is done in Markov State modeling [49], or mean first passages times [50]. However, all three types of matrices, i.e. rate matrices, transition probability matrices and mean first passage time matrices, can be transformed into each other [50]. Hence, although the TN approach seems to focus on one special minimum energy pathway it includes, in principle, the information for all other paths as well.

A challenging aspect in calculating TNs is the exponential increase of stationary points (and thus MEP calculations) on the energy surface with increasing system size [51]. In the case of proton translocations through proton transfer channels, the number of degrees of freedom (DOFs) to sample, corresponding to critical channel residues and intra-channel water molecules, is already problematic. However, investigations in *CcO* as well as *NADH dehydrogenase* furthermore suggest, that the environment of the proton transfer channel is able to affect intra-channel properties [15, 17, 18]. Hence, thorough investigations of the proton translocation should also include the channel environment. Coupling the TN approach with an MD-based sampling of the positions and orientations of the water molecules inside the channel [47] reduces the TN calculation costs significantly. Still, a direct inclusion of the DOFs of the channel environment in the sampling of proton transfer pathways is infeasible. Therefore, the channel environment needs to be considered indirectly, i.e. several TN calculations need to be performed for varying configurations of the channel environment, to gain a comprehensive understanding of this exceptionally important process.

Here, we present a method, which characterizes TNs for different configurations of the unsampled channel environment by determining important graph theoretical properties, i.e. the minimum spanning tree (MST) and minimax best pathway (MBP), defined in the methods section, using an extensive coarse-graining of the underlying transition networks and the MST of an existing, complete TN calculation as initial guess. Thereby the calculation costs are reduced significantly, while important network properties are maintained, e.g. the maximal barrier of the transition [52]. To validate the novel method we used the same channel-like proton transfer system as in *Reidelbach et al* [47]. Here, however, we introduced a fixed point charge in the vicinity of the channel, representing the unsampled channel environment. Several MSTs or MBPs were determined for various translocations, increases or decreases of the point charge, and compared to the MSTs or MBPs of the respective complete TN calculations. To distinguish the MST or MBP characterization of unknown TNs (as introduced before) from complete TN calculations, the approach presented in this work is termed TN prediction.

## Materials and methods

**Fig 1:**
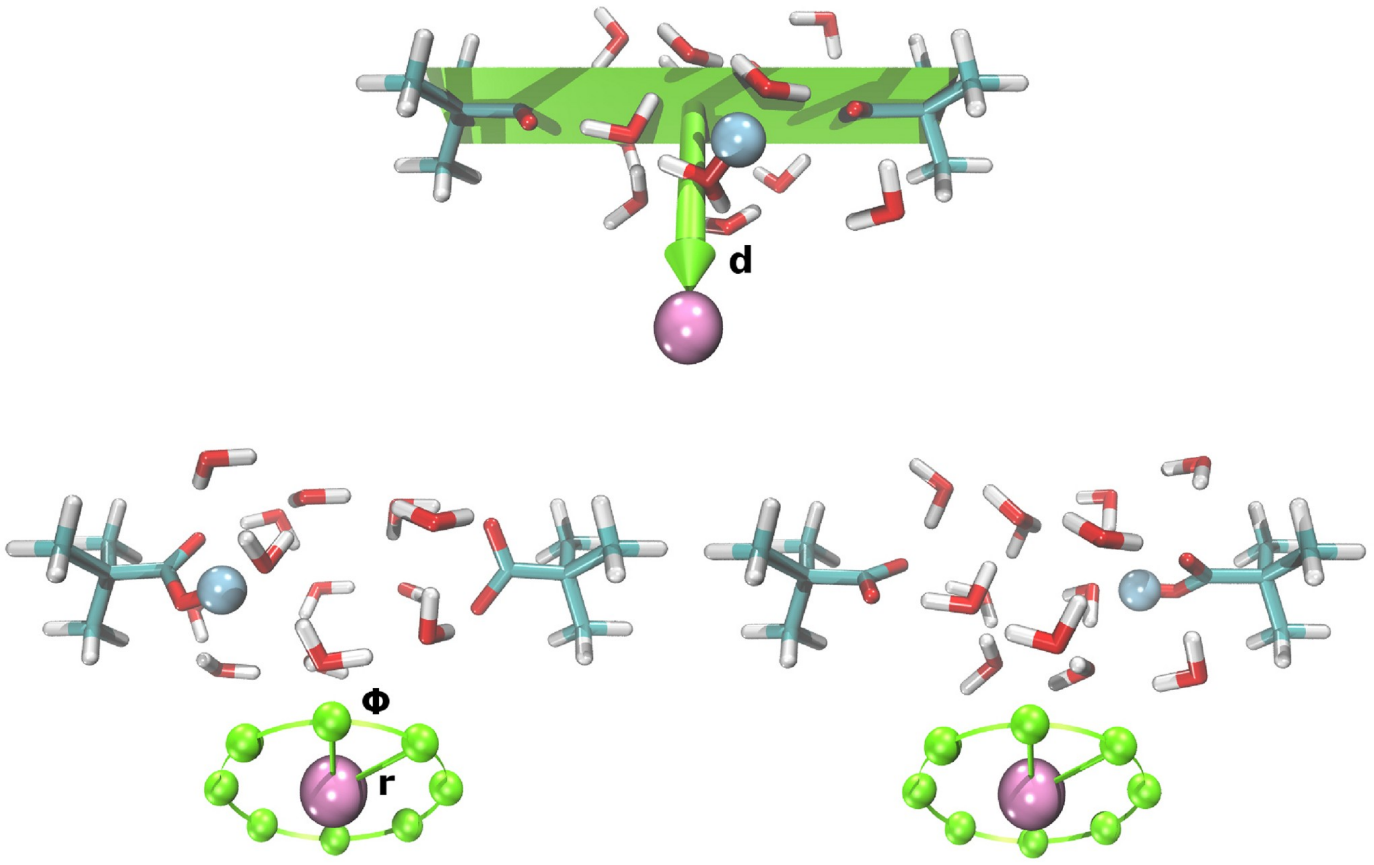
Model system for proton transfer. Model system for calculating proton transfer pathways with an additional point charge (pink sphere). Top: plane containing the cylindrical axis and the orthogonal verctor **d** (|**d**| = 6.0 Å) to locate the initial position of the additional point charge, bottom left: reactant state, bottom right: product state. The blue sphere highlights an excess proton located on the left or right carboxyl group corresponding to the reactant or product state of the proton transfer reaction. Circular translocations of the additional point charge, parallel to the plane with |**r**| = 0.5 Å, 1.0 Å, or 2.0 Å and *ϕ* = 0°, 45°, 90°, 135°, 180°, 225°, 270°, or 315°, are indicated by green spheres. Charge increases or decreases are not depcited.

Recently, we investigated the proton transfer through a cylinder filled with thirteen water molecules and one excess proton. Top and bottom of the cylinder were formed by two stationary t-butyl structures, while a harmonic potential, setting on at 3.0 Å away from the cylindrical axis with a force constant of 500 kcal/mol/Å^2^, modeled the walls. Attached to the central carbon atom of either t-butyl structure was a carboxyl group pointing inside the cylinder. Hence, the model system resembled a water-filled channel in a protein connecting two aspartate or glutamate-like side chains [47]. In the current study we extended our model by introducing a fixed point charge in the vicinity of the cylinder, 6 Å away from the midpoint of the cylindrical axis (cf Supplementary Information for a detailed description of the inital position). Value and position of the point charge were constant within individual TN calculations, corresponding to one TN for each point charge position or value. The initial value of the point charge was set to *q* = 0.050. Other configurations of the point charge were achieved by circular translocations around the initial position with |**r**| = 0.5 Å, 1.0 Å, or 2.0 Å and *ϕ* = 0°, 45°, 90°, 135°, 180°, 225°, 270°, or 315°, parallel to a plane containing the cylindrical axis (cf Fig 1), charge decreases down to *q* = 0.000 (actually *q* = 0.000001 to keep the point charge included in the QM/MM setup) in steps of Δ*q* = 0.010 or charge increases up to *q* = 0.100 in steps of Δ*q* = 0.001 from *q* = 0.050 to *q* = 0.060, steps of Δ*q* = 0.002 from *q* = 0.060 to *q* = 0.070 and steps of Δ*q* = 0.005 from *q* = 0.070 to *q* = 0.100, giving 51 configurations overall.

In a first step, we performed 51 complete TN calculations, starting from two configurations with the excess proton located at either of the carboxyl groups, i.e. the reactant and product state of the overall proton transfer reaction. To construct the TNs we sampled different protonation states and side chain conformations of the carboxylated t-butyl structures. Therefore, we placed the excess proton on either water molecule or on one of the carboxyl groups and rotated the side chain dihedral angles in 45° steps, giving 1088 initial states. The initial state set was then subjected to a quantum mechanics/molecular mechanics (QM/MM) energy minimization with a convergence criterion of 0.001 kcal/mol/Å, in which the water-filled cylinder and the additional point charge represented the QM and MM part, respectively. As energy functions we used the semi-empirical OM2 method [53] for the QM part and CHARMM27 [54] for the MM part. Both parts were coupled electrostatically, thus allowed the MM point charge to polarize the QM electron density, while van der Waals interactions between the QM and MM part were modeled by the MM force field. Following the minimization, the optimized states were classified with respect to the overall reactant state, regarding their side chain dihedral angles, protonation state and water pattern, which gave the nodes of the TN, i.e. a set of unique states representing the branching points of the TN. The side chain dihedral angles and the protonation state were assigned according to the sampling of the initial states, e.g. a 45° rotation of either dihedral angle compared to the overall reactant state or a change in the protonation state compared to the overall reactant state results in a node distinct from the overall reactant state. The motion of the water molecules was not considered in the initial sampling. Still, the pattern of all water molecules in the model channel, defined by their positions and orientations, was included in the state assignment to account for the highly dynamic nature of the water molecules. The minimal difference between two unique water patterns was the translation of a single water molecule by 2 Å in x, y, or z-direction compared to the overall reactant state or the rotation of a single water molecule by 22.5° around the x, y, or z-axis compared to the overall reactant state. To avoid chemically equal nodes, which only differ in their atom labels, e.g. due to a 180° rotation of a deprotonated carboxyl group or due to water molecules switching positions, an excessive atom re-labeling is performed with respect to the overall reactant state using an implementation of the *Hungarian algorithm* [46]. Once the node set of the TN was determined, transitions between selected pairs of nodes, i.e. nodes which differed in each DOF by at most one step, were computed using the CPR method [27], which provided the edges of the TN, i.e. the node connections of the TN. Note that this still allows a transition of a proton along several water molecules simultaneously since the difference in DOFs is evaluated for the proton position to have changed but not by how much. As edge weights we used the energy of the highest transition state in between adjacent nodes relative to the overall reactant state. Nodes and edges were then combined to form a weighted, undirected graph. Finally, the MBP, i.e. the pathway with the lowest highest transition state energy (termed ω* in this work), connecting the reactant and product state of the overall proton transfer reaction was computed using Dijkstra’s algorithm [55].

**Fig 2:**
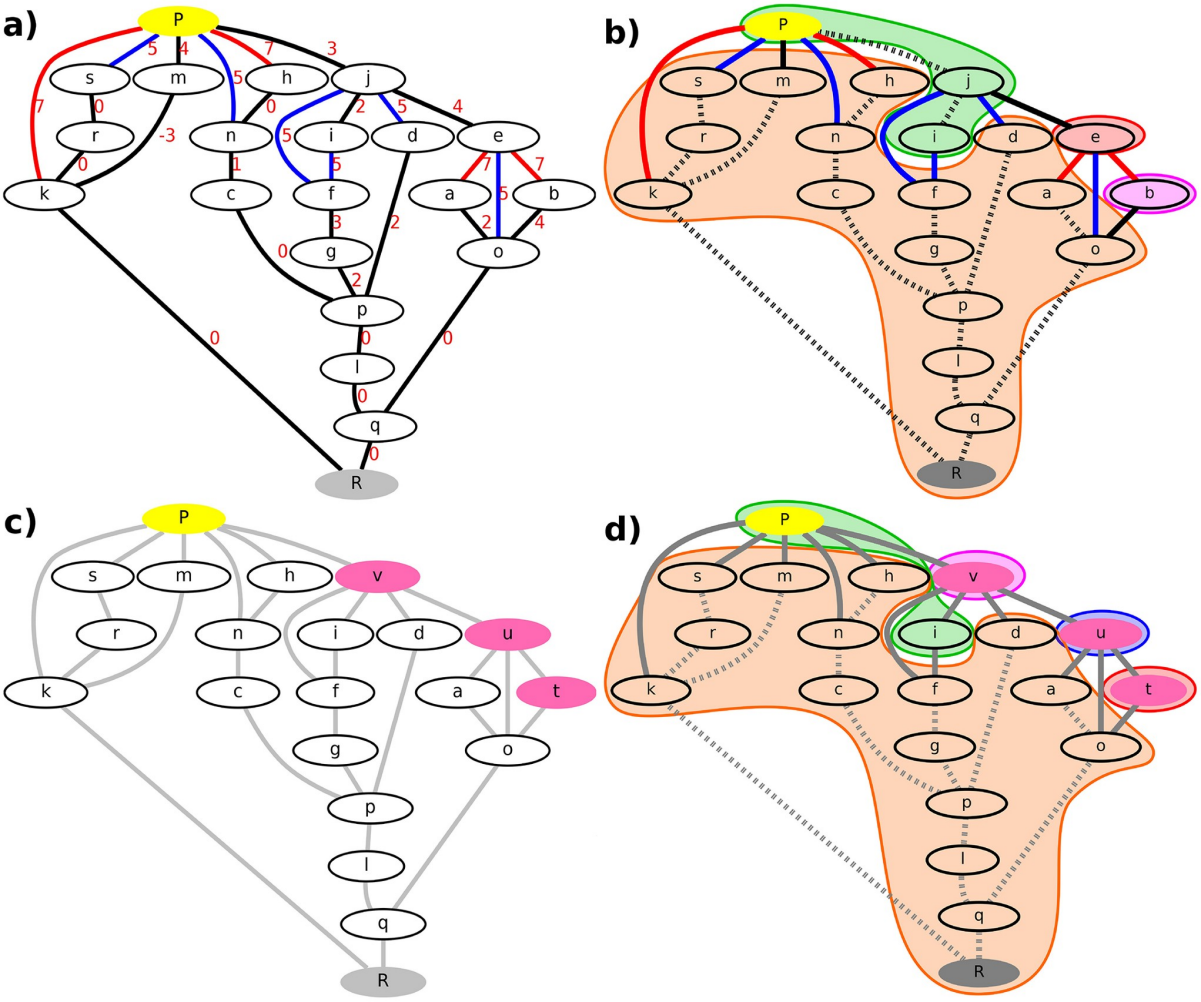
Steps of the TN prediction method. a) Initial TN (combined TN from *Reidelbach et al* [47]) containing 21 nodes and 29 edges. Nodes are shown as ellipses, edges are shown as lines. The reactant state is highlighted in gray, the product state in yellow, labelled by R and P. Intermediate nodes are labelled from a to s. A complete description of either node regarding the side chain dihedral angles of the carboxylated t-butyl structures, the water pattern, and the protonation state can be found in S1 Table. Red numbers represent the energy of the maximal transition state along the edge used as edge weight. All energies are in kcal/mol, relative to the overall reactant energy and rounded to integer values. Edges in black represent the MST, edges in blue represent high sensitive non-MST edges and edges in red represent low sensitive non-MST edges. b) Coarse-grained representation of the initial TN containing 4 coarse-grained nodes, represented by the orange, green, magenta, and red shaded areas, and 29 edges. Dashed lines represent negligible edges connecting nodes within the same coarse-grained node, solid lines represent edges connecting nodes within distinct coarse-grained nodes. Edge weights are not shown for a better visualization. c) Perturbed TN containing 21 nodes and 29 edges. Nodes b, e, and j are replaced by the nodes t, u, and v, highlighted in pink. Gray lines indicate edges to be calculated in an all-node TN. d) Coarse-grained representation of the perturbed TN based on the initial coarse-graining of the initial TN containing 5 coarse-grained nodes, represented by the orange, green, magenta, blue, and red shaded areas, and 29 edges. Dashed lines represent negligible edges, solid lines represent edges to be calculated.

In a second step, we performed 50 TN predictions using the previously determined complete TN with the point charge in its initial configuration, the initial TN, as starting point for the TN predictions of all other configurations, termed here the perturbed TNs. To characterize the initial TN we determined its MST, i.e. edge subset connecting all nodes without cycles and minimal edge weight sum [56], using Kruskal’s [57] algorithm. The MST provides the MBPs between all pairs of nodes and is unique for TNs with distinct edge weights. In principle, each non-MST edge could be part of the MST if its weight is reduced appropriately. Non-MST edges for which a small weight reduction is already sufficient to alter the MST are called high sensitive edges, low sensitive edges are identified accordingly (cf Fig 2, top left). We calculated the sensitivity of all non-MST edges, which allowed us to order the non-MST edges from high to low sensitivity. The MST of the initial TN is later on used as initial guess for the MST or MBP of the perturbed TNs, while the ordered initial non-MST edge lists are used for the refinement of the initial guess.

Once the initial MST and ordered initial non-MST edge list were determined, we coarse-grained the initial TN (initial coarse-graining). In contrast to other coarse-graining techniques, the performed clustering is based on transition barriers, using ω* as upper bound, instead of a (usual) structure-related clustering [58]. Therefore, we determined the eigenvectors of the *Laplacian matrix*, **L**, corresponding to the initial TN,

$$\begin{array}{c} L=D-A^{*}, \end{array}$$ (1)

where **D** is a diagonal matrix containing the degrees of all nodes of the initial TN and A* is a special adjacency matrix of the initial TN with

$$\begin{array}{c} a_{ij}=\{\begin{array}{c} 0\;\text{if}\;\omega_{ij}>\omega^{*}\\ 1\;\text{if}\;\omega_{ij}<\omega^{*} \end{array}, \end{array}$$ (2)

to determine the connected components and thus the coarse-grained nodes. By that, the initial MBP is for example reduced to a path with two coarse-grained nodes (each containing at least one original node) connected by an edge with ω* as weight (cf Fig 2, top right). These coarse-grained nodes, however, cannot be understood as single representative conformations, e.g. average conformations of original nodes or original nodes most similar to the average conformations, because these representative conformations are in most cases no local minima conformations, which is required by the CPR algorithm, or they suffer from the initial guess-path problem. Instead, they need to be interpreted as sets of original nodes between which barrier-free transitions (compared to ω*) are possible. Hence, the coarse-graining step is not actually reducing the number of nodes to look at, but the number of edges, i.e. all edges connecting nodes within the same coarse-grained node can be excluded from further investigations. Thereby, we reduced the sets of edges representing the initial MST and ordered initial non-MST edge list to those edges connecting distinct coarse-grained nodes only.

Following the analysis of the initial TN, we redid the sampling of the initial states, the minimizations and the node pairing in the perturbed systems, according to the same criteria of changes in DOFs between pairs of nodes as in the initial TN. Thereby, we calculated all nodes of the perturbed node sets and identified all edges to be calculated for the perturbed edge sets, thus receiving a comprehensive picture of the topology of the perturbed TNs. Furthermore, we assumed that the initial coarse-graining is still valid, thus disregarding all edges connecting nodes within the same coarse-grained node in the perturbed edge sets (cf Fig 2, bottom). The coarse-grained initial MST and coarse-grained ordered initial non-MST edge list were then adjusted to the perturbed TNs: All edges present in the initial edge set but absent in the perturbed edge sets were removed from the edge sets representing the coarse-grained initial MST and coarse-grained ordered initial non-MST edge list, on the other hand, edges present in the perturbed edge sets but absent in the initial edge set were added to the edge set representing the coarse-grained ordered initial non-MST edge list. To integrate these edges properly into the sensitivity ranking the inverse of the maximal transtion barrier of the MBP connecting the nodes in the initial TN was used as sensitivity value for these new edges. The sensitivity value for edges connecting nodes not present in the initial node set was set to zero. If several edges were assigned the same sensitivity value, they were ordered randomly. Hence, the coarse-grained initial non-MST edge list is ordered from high to low sensitivity values with a non-deterministic order for edges with equal sensitivity values.

**Fig 3:**
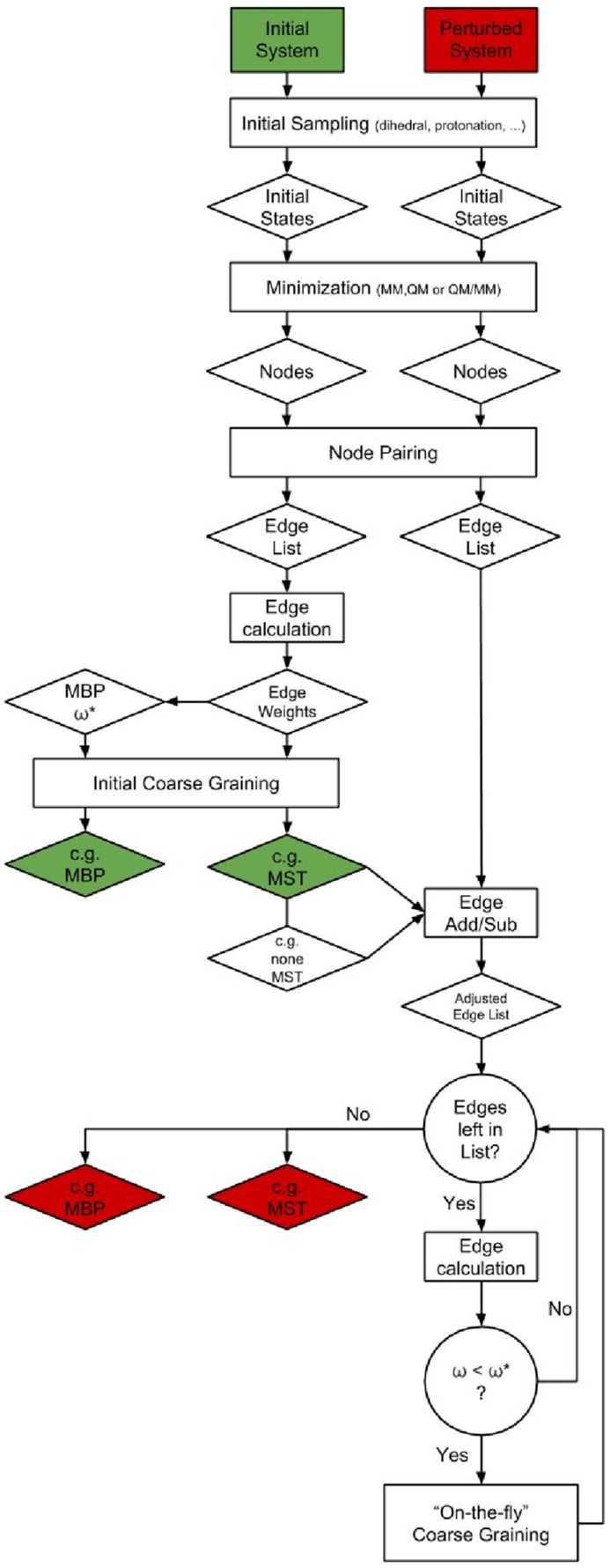
Flow chart of the TN prediction method. Flow chart representing the TN prediction method starting from an initial TN calculation. (Abbreviations are explained in the text).

The remaining coarse-grained initial MST edges were then calculated in the perturbed TNs. In few cases these calculations were already sufficient to obtain the coarse-grained MST or coarse-grained MBP of the perturbed TNs. If that was not the case we calculated additional non-MST edges from the coarse-grained ordered initial non-MST edge lists in the order from high to low sensitivity values. The calculation of additional edges allowed a further coarse-graining of the perturbed TNs (“on-the-fly” coarse-graining), i.e. merging coarse-grained nodes if connected by an additional edge with edge weight below ω*, thus reducing the coarse-grained ordered initial non-MST edge lists even further along the edge calculation. The calculations were stopped once the coarse-grained MSTs or coarse-grained MBPs of the perturbed TNs resembled the results from the complete TN calculations or once no further edges were left to calculate. In Fig 3 the TN prediction method is summarized.

In a third and final step, we tested our TN prediction method for an extended set of perturbations using each of the previously determined complete TNs as initial TN for the prediction of the MST or MBP of all other TNs, yielding 702 and 600 TN predictions for the increase or decrease of the additional point charge or the translocation around its initial position, respectively.

All energy minimizations and CPR calculations were performed using the CHARMM programme [59] interfaced to MNDO [60]. The generation of the initial states, the node assignment, the neighbor search as well as the compilation and analysis of the TNs were performed with our own java code and java libraries from *Noe et al* [45]. The determination of the MSTs and the sensitivity analysis, the initial coarse-graining, and the “on-the-fly” coarse-graining during the TN prediction were performed with our own python code.

## Results and discussion

### Complete transition network calculations

**Fig 4:**
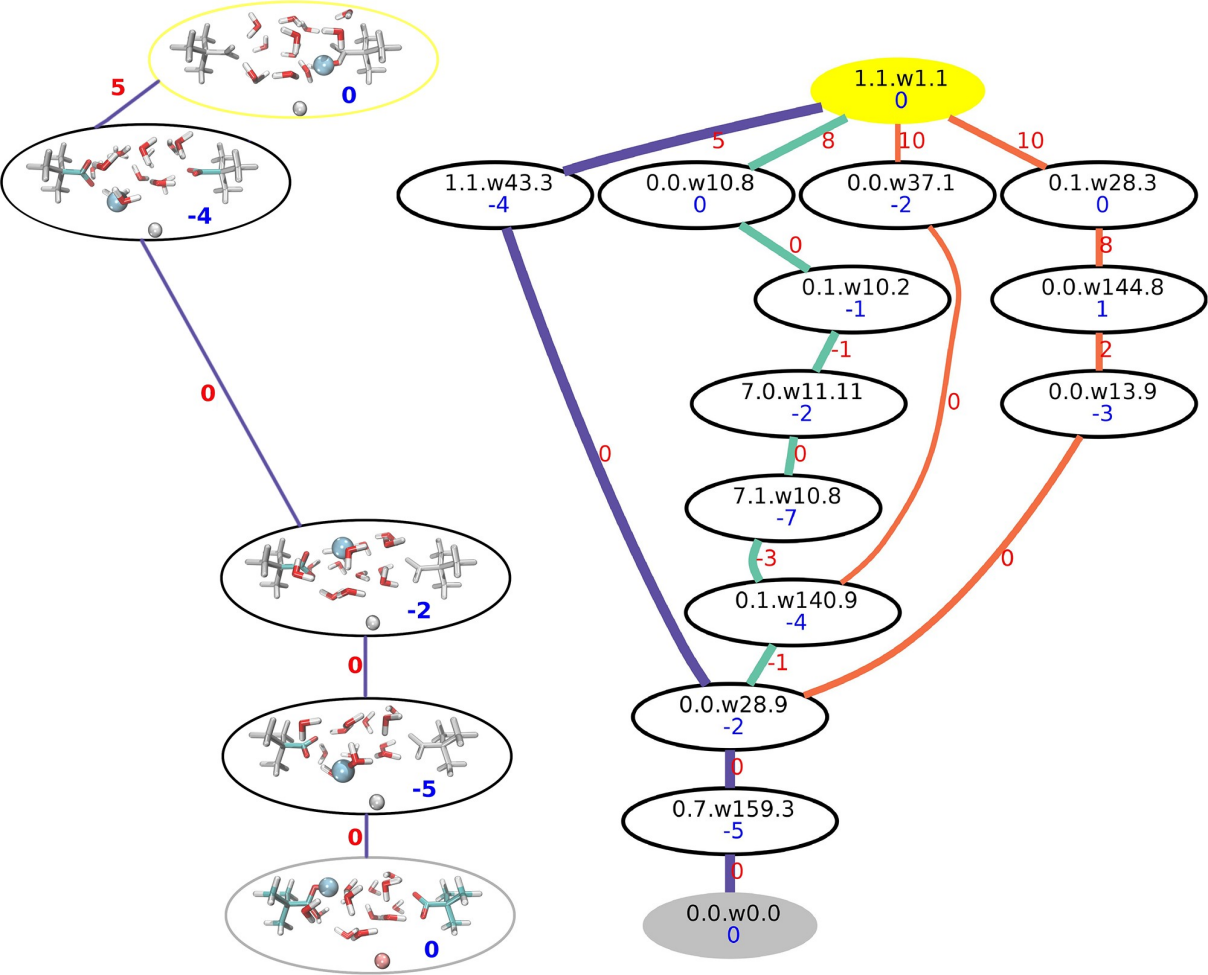
Initial TN. Right: Initial TN of a 13 water model system with additional point charge from a complete TN calculation. The nodes are shown as ellipses, labelled according to the side chain dihedral angles of the carboxylated t-butyl structures, the water pattern and the protonation state (SC0.SC1.Wi.P). The reactant state is shown in gray, the product state in yellow. Edges are shown as lines. Blue numbers represent the energy of the nodes, red numbers the energy of the maximal transition state along the edge used as edge weight. All energies are in kcal/mol, relative to the overall reactant energy and rounded to integer values. The MBP is highlighted in indigo, the next best paths are shown in turquoise and orange. Left: Detailed representation of the best pathway. The reactant state is shown in gray, the product state in yellow. Edges are shown as lines. Blue numbers represent the energy of the nodes, red numbers the energy of the maximal transition state along the edge used as edge weight. All energies are in kcal/mol, relative to the overall reactant energy and rounded to integer values. Colored structure elements indicate structural changes along the transition according to the assignment of the TN calculation.

In a first step 51 complete TNs were calculated. These were the initial TN, 26 TNs with an increased or decreased value of the additional point charge and 24 TNs with charge translocations around the initial position. Here, only the results for the initial TN are presented in detail (a detailed description of all 51 TN calculations is given in S2 Table and S1 to S9 Figs). The initial TN calculation resulted in a network with 252 nodes connected by 20316 edges. The MBP connecting the reactant and product state of the overall proton transfer reaction contains three intermediate nodes and a maximal transition barrier of 5 kcal/mol. This pathway involves the re-arrangement of both side chain dihedral angles, the protonation state and the water pattern. Interestingly, the change in the side chain dihedral angle of the second carboxylated t-butyl structure and protonation state is not gradually. Instead, back transitions to the overall reactant state or previous intermediate node are observed, e.g. a counterclockwise rotation of ≈ 45° (transition from 0 to 7) followed by a clockwise rotation of ≈90° in two steps (transitions from 7 to 0 and 0 to 1) for the side chain dihedral angle of the second carboxylated t-butyl structure (cf. Fig 4, indigo pathway). The next best proton transfer pathways have transition barriers of 8 and 10 kcal/mol, respectively. The direct TN calculation without an external point charge [47] gives the same maximal transition barrier for the MBP. However, the intermediate states involved differ, simply due to the fact that the conformation of the reactant and product states differ. Nevertheless, structural elements, in terms of visited protonation sites, of the second best proton transfer pathway resemble the pathway previously reported.

The increase, decrease and translocation of the external point charge affects the MBP properties. For point charge increases or decreases for example the maximal transition barrier of the MBP varies between 3 and 9 kcal/mol with an average maximal transition barrier of the MBPs of 5 ± 1.5 kcal/mol (5 ± 1.5 kcal/mol for charge translocations), while the number of intermediate nodes varies between 1 and 7 nodes with an average number of intermediate nodes of 5 ± 1 (5 ± 1 for charge translocations). Furthermore, the general network topology of either TN is affected to a large extent. In case of charge translocations for example 33 to 69% of the nodes and 52 to 87% of the edges present in the initial TN do not exist in the perturbed TNs following the charge translocations (41 to 60% of the nodes and 58 to 80% of the edges for point charge increases or decreases). On the other hand, 30 to 70% of the nodes and 50 to 87% of the edges present in perturbed TNs do not exist in the initial TN (38 to 68% of the nodes and 56 to 82% of the edges for point charge increases or decreases). Hence, in most cases the perturbed TNs contain more “unknown” topological features than “known”. Thus, for an efficient determination of the coarse-grained perturbed MSTs or MBPs the information provided by the initial TN is in most cases not sufficient. Instead, further information about the perturbed TNs need to be acquired while calculating them. In our TN prediction method this fact is acknowledged by the “on-the-fly” coarse-graining step.

### Transition network predictions

The TN prediction method proposed in this paper contains three fundamental steps. These are:

1. The usage of the MST of the initial TN as initial guess for the MST of the perturbed TNs followed by an ordered non-MST edge calculation according to pre-determined edge sensitivity values.
2. The initial coarse-graining using information from the initial TN.
3. The “on-the-fly” coarse-graining using information from the previous edge calculations for the next edge calculations.

**Fig 5:**
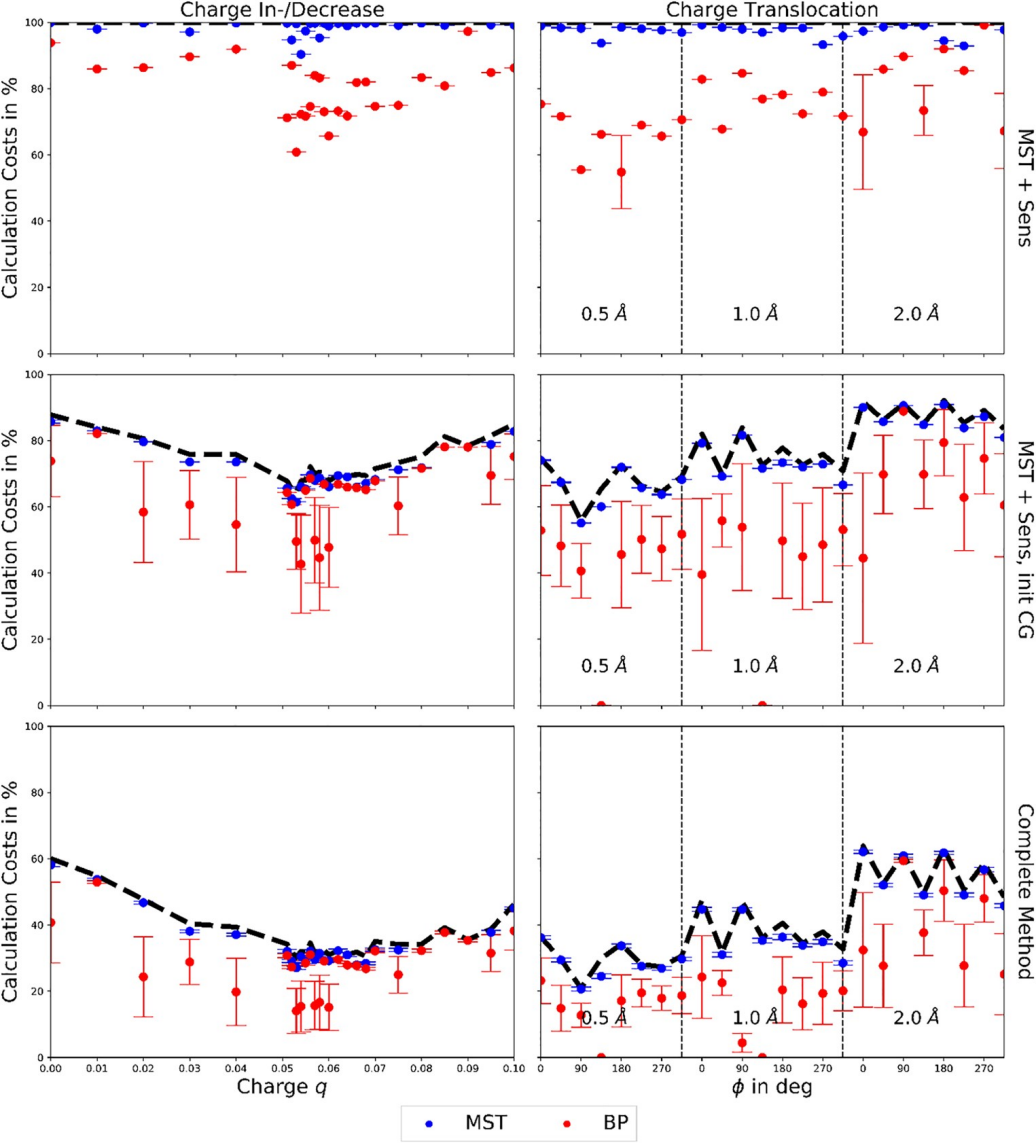
Average calculation cost of MST and MBP predictions. Average calculation costs (in % of the average length of the ordered non-MST edge lists without coarse-graining) for the (all-node or coarse-grained) prediction of the perturbed MST (blue) and MBP (red) from 1000 prediction runs per perturbation. Perturbations: charge in-/decrease (left column), charge translocation (right column). Top row: calculation by MST and sensitivity (all-node results), middle row: calculation by MST, sensitivity and initial coarse-graining (coarse-grained results), bottom row: complete method using the previous steps and the “on-the-fly” coarse-graining (coarse-grained results). The average length (as before in % of the average length of the ordered non-MST edge lists without coarse-graining) of the ordered non-MST edge lists (all-node or coarse-grained) used for the calculations is depicted in black, as dashed line for a better visualization.

To demonstrate the cost reductive effect of either step Fig 5 displays the costs of the edge calculations, i.e. the number of essential edge calculations compared to the size of the perturbed edge sets in %, for an increase or decrease of the point charge or its translocation around the initial position, when only using the MST and its sensitivity, the MST, its sensitivity and the initial coarse-graining, or the complete method using the previous steps and the “on-the-fly” coarse-graining. All calculations were performed 1000 times per perturbation scenario to study the effect of the non-deterministically ordered edges (due to equal sensitivity values) within the all-node or coarse-grained ordered non-MST edge lists and were stopped once the coarse-grained MSTs or coarse-grained MBPs of the perturbed TNs resembled the results from the complete TN calculations or once no further edges were left to calculate.

The determination of the perturbed MSTs required average calculations of 99%, 71%, or 35% of the non-MST edge lists for charge increases or decreases and 97%, 75%, or 40% for charge translocations, using the MST and its sensitivity, the MST, its sensitivity and the initial coarse-graining, or the complete method using the previous steps and the “on-the-fly” coarse-graining, respectively. The calculation costs for both types of perturbation do not depend on the severity of the perturbation, when performing calculations without coarse-graining. However, when applying the initial coarse-graining or the initial and “on-the-fly” coarse-graining the calculation costs depend on the severity of the perturbation, e.g. costs for translocations of 0.5 Å < costs for translocations of 1.0 Å < costs for translocations of 2.0 Å. Interestingly, the calculation costs are not symmetric for charge increases and decreases, revealing a calculation cost difference of 12% for Δ*q* = ± 0.050 when using the initial and “on-the-fly” coarse-graining. The prediction of the perturbed MSTs for charge increases and decreases or charge translocations around the initial position is based on the MST of the unperturbed TN, used as initial guess, giving rise to a possible bias of the predicted, perturbed MSTs towards the initial guess. However, a comparison of the initial guess MST with the predicted, perturbed MSTs reveals a maximal edge similarity of the MSTs of only 10% and 20% (on average 5 ± 3% and 10 ± 6%) for charge increases or decreases and charge translocations around the initial position, respectively. On the other hand, the node similarity of the MSTs equals, obviously, the node similarity of the TNs stated before. Hence, the initial guess MST and predicted, perturbed MSTs are sufficiently different to indicate a bias-free prediction. Furthermore, the TN prediction method is able to reproduce all MSTs from the complete, perturbed TN calculations (without any initial guess), indicating once more a bias-free prediction.

The determination of the perturbed MBPs required average calculations of 80%, 64%, or 28% of the non-MST edge lists for charge increases or decreases and 75%, 51%, or 23% for charge translocations, using the MST and its sensitivity, the MST, its sensitivity and the initial coarse-graining, or the complete method using the previous steps and the “on-the-fly” coarse-graining, respectively. The calculation costs for the MBPs depend on the severity of the perturbation. For charge translocations of |**r**| = 0.5 Å or 1.0 Å and *ϕ* = 135° the calculation of the initial MST edges was already sufficient to determine the perturbed coarse-grained MBPs. In all cases the calculation of the perturbed MBPs required less edge calculations than the calculation of the perturbed MSTs, which is trivial since the MBP is a subset of the MST.

The TN prediction method reduces the costs of the MST or MBP determination by coarse-graining, and thus reducing, the ordered non-MST edge lists (cf Fig 5 dashed black lines). A complete calculation of the coarse-grained non-MST edge lists guarantees the most accurate determination of the perturbed MSTs or MBPs (at least within the error related to the coarse-graining, discussed later on) and cost reductions of 40 up to 80% (compared to the edge calculation costs of the respective complete TN calculations). In principle, further cost reductions are possible, at least if one is only interested in the coarse-grained perturbed MBPs (cf Fig 5 red dots and previous paragraph). However, defining a lower edge calculation bound providing accurate coarse-grained MBPs is problematic due to high fluctuations (up to ± 17%) associated with the non-deterministic order of edges with equal sensitivity values within the coarse-grained ordered non-MST edge lists used for the predictions. Therefore, a complete calculation of the coarse-grained ordered non-MST edge lists should be performed for all determinations of the perturbed MSTs or MBPs. A further benefit of a complete calculation, compared to a pre-set edge calculation bound, is that the amount of edges to be calculated is flexible, self-regulated by the TN prediction method and constantly adjusted to the requirements of the perturbed TNs.

**Fig 6:**
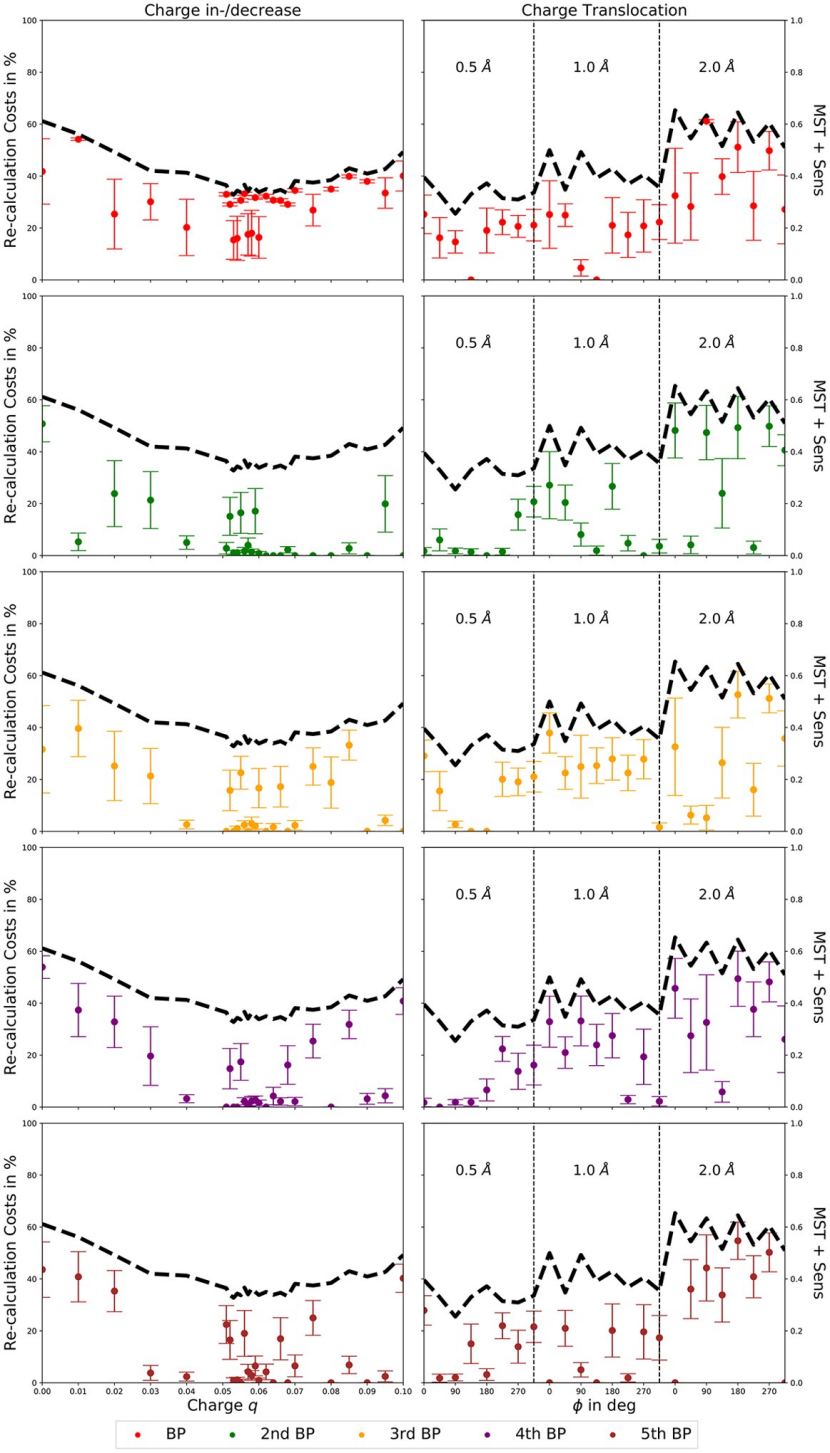
Average calculation cost of next best pathway predictions. Average calculation costs (in % of the average length of the ordered non-MST edge lists without coarse-graining) for the coarse-grained prediction of the perturbed MBP (red), 2nd MBP (green), 3rd MBP (orange), 4th MBP (purple), and 5th MBP (brown) from 1000 prediction runs per perturbation using the MST and its sensitivity, the initial coarse-graining, and the “on-the-fly” coarse-graining. Perturbations: charge in-/decrease (left column), charge translocation (right column). The average length (as before in% of the average length of the ordered non-MST edge lists without coarse-graining) of the coarse-grained, ordered non-MST edge lists used for the calculations is depicted in black, as dashed line for a better visualization.

A single reaction pathway is often not enough to properly describe a chemical reaction [22], e.g. the transfer of protons. Therefore, we determined the perturbed second, third, fourth, and fifth best MBPs, next to the actual MBPs, with our TN prediction method. The results for charge increases or decreases and charge translocations around the initial position, using the MST and its sensitivity, the initial coarse-graining, and the “on-the-fly” coarse-graining, are presented in Fig 6.

The average calculation costs for the prediction of the perturbed second, third, fourth, or fifth best MBPs fluctuate, in most cases, around the calculation costs for the actual perturbed MBPs. Thus, a complete calculation of the coarse-grained non-MST edge lists (dashed black lines), guarantees not only the most accurate determination of the MBPs, but also the most accurate determination of the second, third, fourth, and fifth best MBPs. Thereby, the TN prediction method is able to provide a proper description of chemical reactions in a cost efficient manner.

### Coarse-graining problems

In all perturbation scenarios significant cost reductions were achieved by the initial and “on-the-fly” coarse-graining steps. These steps, however, are also potential sources of error regarding the determined maximal transition barriers of the perturbed MBPs or properties related to the MSTs. Here, we focus on the accuracy of the maximal transition barriers of the perturbed MBPs.

**Fig 7:**
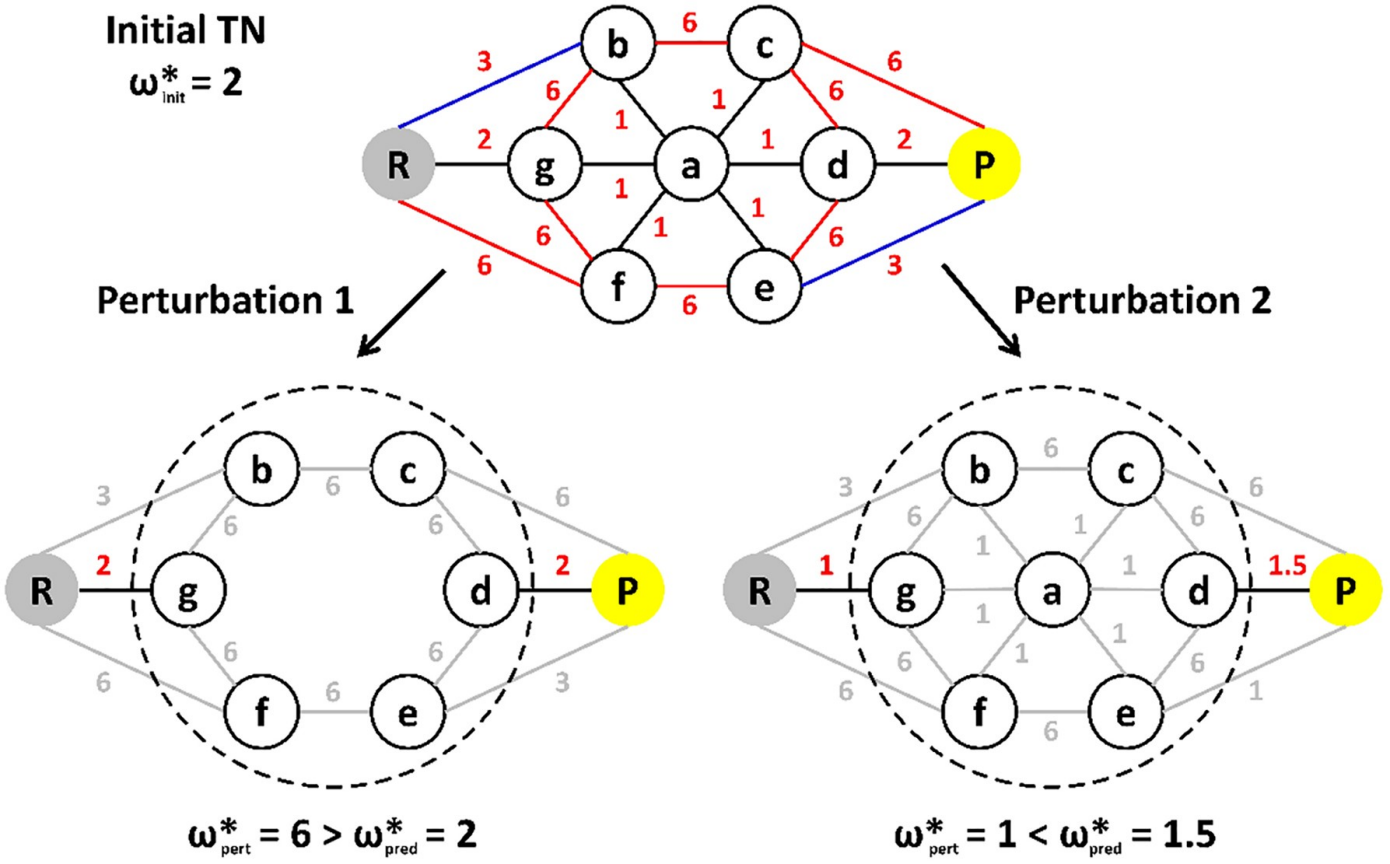
Coarse-graining problems. Initial TN: Nodes are depicted as circles, edges as lines. Reactant and product state are shown in gray and yellow labelled R and P, respectively, other nodes are labelled from a to g. The edge weights are in kcal/mol and shown in red. Perturbed TNs: Node labeling as before, calculated edges are depicted in black, edges to be calculated are depicted in gray. The dashed circles indicate the main coarse-grained node according to the initial coarse-graining. Other coarse-grained nodes are the reactant and product state.

The initial coarse-graining is based on the assumption that the integrity of the coarse-grained nodes is preserved beyond the perturbation, thereby allowing the neglect of edges connecting nodes within the same coarse-grained node from the perturbed edge lists. In principle, node additions or subtractions to or from a coarse-grained node are possible and occur frequently. Still, it is required that all nodes within a coarse-grained node can be reached by crossing barriers below $\omega_{init}^{*}$ only. In Fig 7 “Perturbation 1” is not fulfilling this requirement, i.e. due to the subtraction of node a from the coarse-grained node, barriers of 6 kcal/mol need to be crossed in order to reach every node within the coarse-grained node, while the initial coarse-graining assumes that all nodes can be reached by crossing barriers below 2 kcal/mol. Therefore, the maximal transition barrier of the perturbed MBP would be 6 kcal/mol if all nodes are considered and 2 kcal/mol if the initial coarse-graining is applied. Hence, the initial coarse-graining step is prone to maximal transition barrier underestimations, while overestimations are ruled out.

The “on-the-fly” coarse-graining combines two coarse-grained nodes if they are connected by an edge with edge weight below $\omega_{init}^{*}$, thereby neglecting all further edges connecting the two coarse-grained nodes. This setup ensures an exact refinement of $\omega_{pert}^{*}$ if $\omega_{pert}^{*}\geq \omega_{init}^{*}$. In the opposite case, however, the “on-the-fly” coarse-graining could stop the refinement of $\omega_{pert}^{*}$ too early. A typical situation is displayed in Fig 7 by “Perturbation 2”. The initial coarse-graining provided three coarse-grained nodes. Following the calculation of the coarse-grained initial MST edges in the perturbed system all coarse-grained nodes will be combined by the “on-the-fly” coarse-graining, preventing further edge calculations. Therefore, the maximal transition barrier of the perturbed MBP would be 1 kcal/mol if all nodes are considered and 1.5 kcal/mol if the “on-the-fly” coarse-graining is applied. Hence, the “on-the-fly” coarse-graining step is prone to maximal transition barrier overestimations for situations in which $\omega_{pert}^{*}<\omega_{init}^{*}$, while underestimations are ruled out.

**Fig 8:**
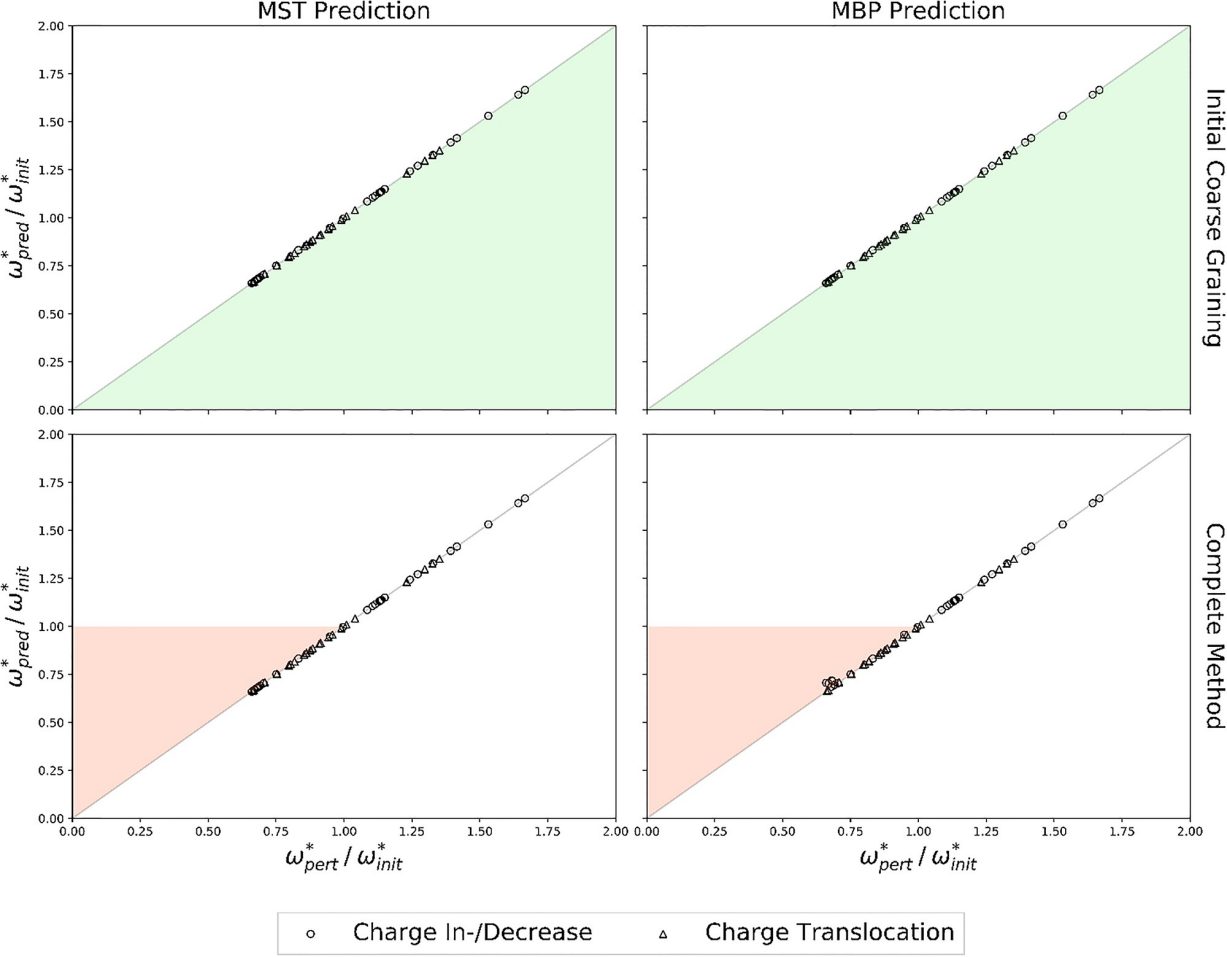
Accuracy of $\omega_{pert}^{*}$ predictions. Comparison of ω* from complete TN calculations ($\omega_{pert}^{*}$) and MST (left column) or MBP (right column) predictions ($\omega_{pred}^{*}$, averaged over 1000 prediction runs per perturbation) following the initial coarse-graining (top row) or the complete method (bottom row). Charge in-/decreases (circles), charge translocations (triangles). Shaded areas indicate potential error regions, underestimations (green), overestimations (red).

To check the accuracy of our predictions we compared the maximal transition barriers of the MBPs of the complete TN calculations ($\omega_{pert}^{*}$) with those accessible after the initial coarse-graining and those finally predicted ($\omega_{pred}^{*}$). The results are summarized in Fig 8.

The maximal transition barriers of the MBPs accessible after the initial coarse-graining are in perfect agreement to the barriers determined by the complete TN calculations, regardless of the type or severity of the perturbation or the ratio $\omega_{pert}^{*}/\omega_{init}^{*}$. Hence, the potential energy surface associated with the system is most likely stabilizing the coarse-grained nodes, rendering perturbations as displayed in Fig 7 by “Perturbation 1” unlikely. The maximal transition barriers of the MBPs derived by the complete method also agree with the barriers determined by the complete TN calculations. However, for situations in which $\omega_{pert}^{*}/\omega_{init}^{*}<1$, i.e. $\omega_{pert}^{*}<\omega_{init}^{*}$, slight inaccuracies can be observed for individual perturbation scenarios. The inaccuracies reported here, however, are well below the RMSDs reported in proton transfer benchmarks for the semi-empirical quantum method OM2 [61]. Hence, the inaccuracies of the TN prediction method are negligible compared to the intrinsic error of the semi-empirical quantum method. Nevertheless, the potential risk of maximal transition barrier over- or underestimations should not be forgotten.

### Influence of the initial transition network

Finally, we tested our TN prediction method for a larger set of perturbations by using each of the complete TNs as initial TN for the prediction of the MST or MBP of all other TNs, thus increasing the amount of TN predictions from 26 and 24 to 702 and 600 for the in-/decrease of the point charge or its translocation around the initial position, respectively. Thereby, we were able to investigate increased perturbation severities, i.e. Δ*q* up to ± 0.100 (instead of ± 0.050) and |**r**| up to 4.0 Å (instead of 2.0 Å), and the influence of the initial TN (and the similarity of the initial and perturbed TN) on the prediction of the MST or MBP of the perturbed TNs.

For the correct prediction of the perturbed MSTs and MBPs different amounts of edges need to be calculated on average. In case of the MSTs not a single correct prediction was observed, when calculating less than ≈ 20% of the perturbed edge sets. On the other hand, calculations of only 5% of the perturbed edge sets provided correctly predicted perturbed MBPs in 16% (charge in-/decrease) or 12% (charge translocation) of all TN predictions. However, once calculations of more than 20% of the perturbed edge sets are performed the increase in correctly predicted perturbed MSTs is higher than the increase in correctly predicted perturbed MBPs, giving correct predictions in 82% and 90% (charge in-/decrease) or 50% and 71% (charge translocation) of all TN predictions for the perturbed MSTs and perturbed MBPs with a calculation of 50% of the perturbed edge sets. With calculations of 60% of the perturbed edge sets the number of correct predictions is above 90% for all cases, except the perturbed MST prediction for charge translocations.

**Fig 9:**
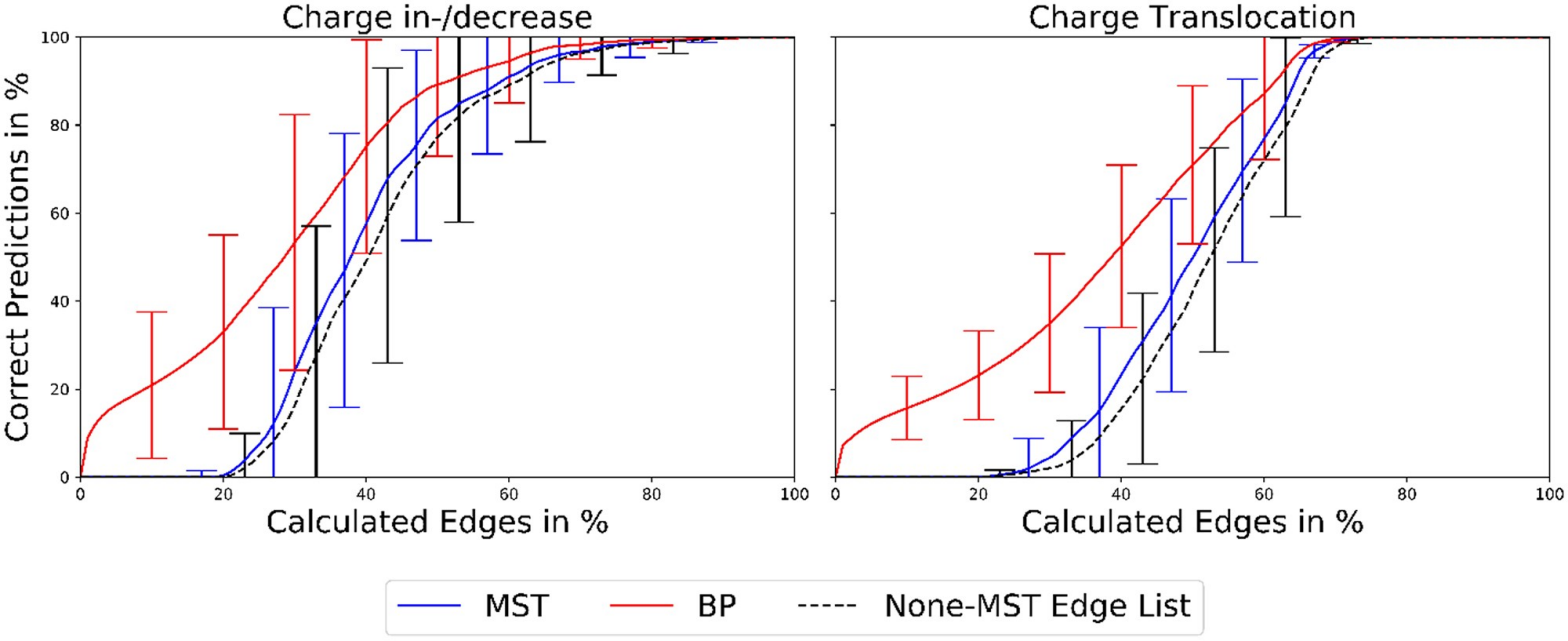
Correct MST and MBP predictions. Correct predictions of the perturbed MSTs (blue) or MBPs (red) for individual numbers of calculated edges averaged over the different initial TNs. The dashed black lines indicate the number of correct MST and MBP predictions by calculating the complete coarse-grained ordered non-MST edge lists averaged over the different initial TNs. Left: charge in-/decrease, right: charge translocation.

The dashed black lines in Fig 9 indicate the number of correct MST and MBP predictions by calculating the complete coarse-grained non-MST edge lists and thus the length of the coarse-grained ordered non-MST edge lists. In principle, the costs for these calculations display the same behavior as those for calculations stopped once the correct perturbed MSTs are reached, shifted by at most 5% to higher edge calculations costs, while the difference to calculations stopped once the correct perturbed MBPs are reached is much larger. In 77% and 42% (or 72%) of all TN predictions the number of edges in the non-MST edge lists was reduced by 50% (or 40%), due to the coarse-graining steps in our TN prediction method, for charge in-/decreases or charge translocations, respectively. Hence, in 77% or 72% of all TN predictions the predicted MSTs or MBPs are unequivocally correct (within the coarse-graining errors described before) when only calculating 50% or 60% of the perturbed edge sets, because there are no further edges left to calculate. As already described before, further edge calculation reductions are possible (cf Fig 9 blue and red lines), but inevitably bear the risk of incorrect predictions of the perturbed MSTs or MBPs, since there are substantial amounts of edges left to be calculated.

Taken as a whole, the number of correct predictions (stopped after the determination of the MSTs or MBPs or the complete calculation of the coarse-grained ordered non-MST edge lists) depends to a large extent on the initial TN or the similarity of the initial and perturbed TN (cf. Fig 9 standard deviations).

**Fig 10:**
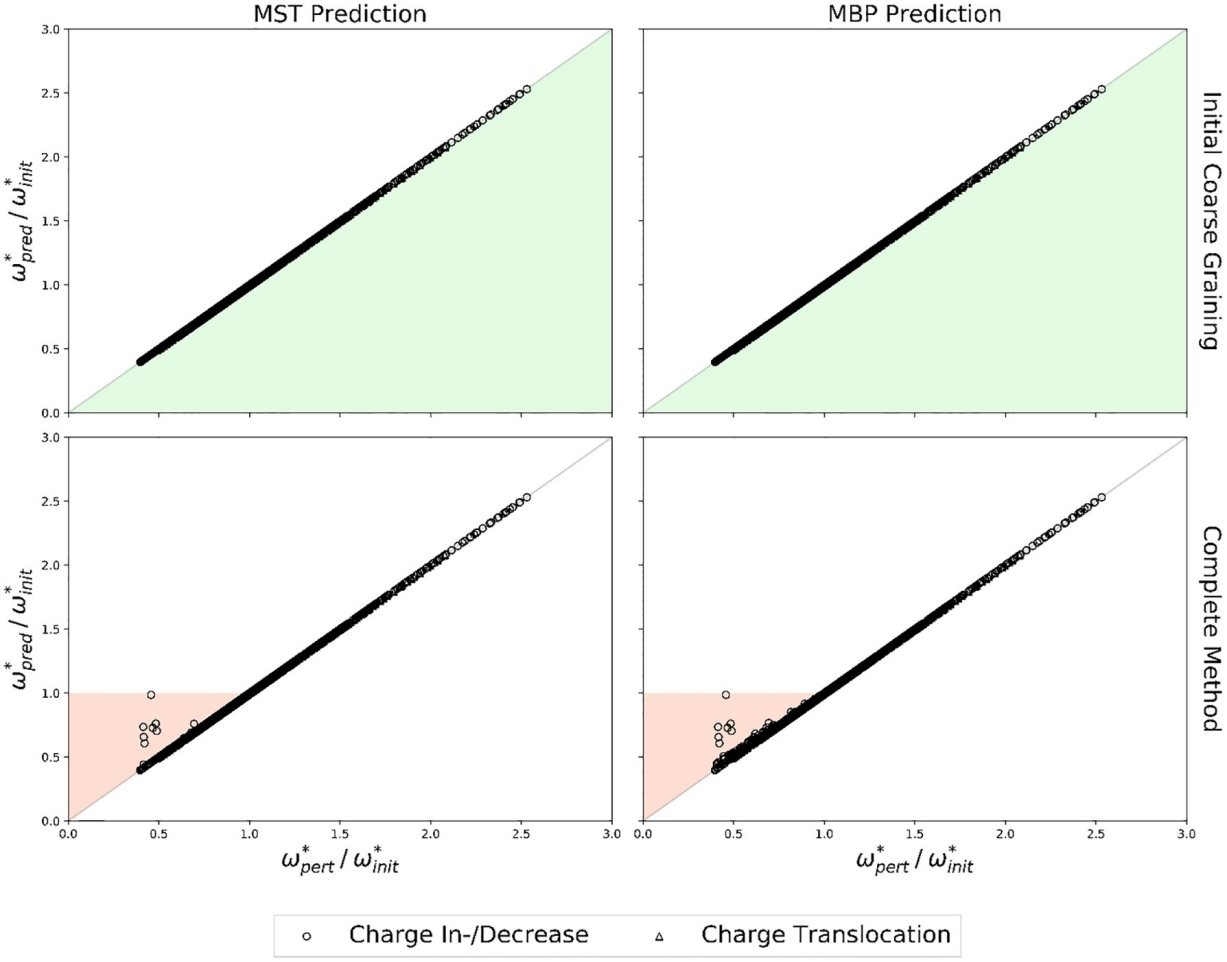
Accuracy of $\omega_{pert}^{*}$ predictions. Comparison of ω* from complete TN calculations ($\omega_{pert}^{*}$) and MST (left column) or MBP (right column) predictions ($\omega_{pred}^{*}$, averaged over 1000 prediction runs per perturbation) following the initial coarse-graining (top row) or the complete method (bottom row). Charge in-/decreases (circles), charge translocations (triangles). Shaded areas indicate potential error regions, underestimations (green), overestimations (red).

Once again we checked the accuracy of our TN predictions by comparing the maximal transition barriers of the MBPs from the complete TN calculations and respective TN predictions. The results are summarized in Fig 10.

As before (cf. Fig 8) not a single maximal transition barrier underestimation was observed following the inital coarse-graining of the perturbed TNs, supporting the hypothesis that the potential energy surface of the particular model studied here is preventing such deviations. The same holds for the maximal transition barriers derived by the complete TN prediction method if $\omega_{pert}^{*}/\omega_{init}^{*}>1$. For the opposite case, however, deviations from the perturbed maximal transition barriers were observed in twelve of all 1302 initial and perturbed TN combinations. Five out of twelve overestimations occured in TN predictions in which the complete TN with *q* = 0.000 was used as initial TN, which is intuitively obvious since the maximal transition barrier of the complete TN with *q* = 0.000 is the highest with 9 kcal/mol and thus provides the maximal range for overestimations. On the other hand, eleven out of twelve overestimations occurred in TN predictions in which the perturbed TNs displayed the lowest maximal transition barrier of 3 kcal/mol (4 kcal/mol for the remaining one), once again providing the maximal range for overestimations. Hence, further investigations might be necessary for situations in which the perturbed maximal transition barriers are lower than the initial ones.

Overall complete calculations of the coarse-grained non-MST edge lists provide significant cost reductions paired with accurate predictions of the perturbed maximal transition barriers.

## Conclusion

The TN prediction method proposed in this paper (summarized in Fig 3) characterizes perturbed TNs by determining their MSTs or MBPs on a coarse-grained level using the MST of an existing, complete TN as initial guess. The costs for a TN prediction are flexible, self-regulated by the TN prediction method and constantly adjusted to the requirements of the perturbed TNs. Thereby significant cost reductions of up to 80% (compared to complete TN calculations) were achieved in a small model system resembling a water filled proton transfer channel for various perturbations of a point charge in the vicinity of the channel. The accuracy of the TN prediction method was tested for the determined maximal transition barriers of the perturbed MBPs, showing for the most part only inaccuracies which were well below the intrinsic error of the semi-empiric calculation method. In few cases more pronounced deviations were observed, rendering further investigations of the perturbed TNs a necessity if the perturbed maximal transition barrier is lower than the initial one.

In principle the TN prediction method proposed in this paper is an extension of Boruvka’s algorithm [62] used for the determination of MSTs. Here, the MST is determined iteratively by coarse-graining the TN until it contains a single node only. Therefore, in every iteration cycle the edges with minimum weight incident to each node are determined and added to the MST, while the TN is coarse-grained along the edges with minimum weight and self loops and multiple edges between pairs of nodes are eliminated (except for the edge with minimum weight). The TN prediction method employs the same concept to determine perturbed MSTs and MBPs when performing the “on-the-fly” coarse-graining. However, it is not possible to use edges with minimum weight incident to each node only, since the weights of all edges are not known a priori. Furthermore, it is not possible to eliminate all multiple edges between pairs of coarse-grained nodes due to peculiarities of the MEP calculation. To compensate for all these drawbacks, an excessive pre-processing, i.e. sensitivity analysis and initial coarse-graining, is performed in a different (but overall similar) TN. Thereby, it is possible to accurately predict the MST and MBP of a perturbed TN, when only calculating a fraction of the perturbed edge set.

## Supporting information

S1 Fig Perturbed TNs with decreased point charge. *q* = 0.000 to *q* = 0.040.
(PDF)

S2 Fig Perturbed TNs with increased point charge. *q* = 0.051 to *q* = 0.055.
(PDF)

S3 Fig Perturbed TNs with increased point charge. *q* = 0.056 to *q* = 0.060.
(PDF)

S4 Fig Perturbed TNs with increased point charge. *q* = 0.060 to *q* = 0.070.
(PDF)

S5 Fig Perturbed TNs with increased point charge. *q* = 0.075 to *q* = 0.100.
(PDF)

S6 Fig Perturbed TNs with translocated point charge. |**r**| = 0.5 with *ϕ* = 0°, 45°, 90°, 135°, 180°, and 225°.
(PDF)

S7 Fig Perturbed TNs with translocated point charge. |**r**| = 0.5 with *ϕ* = 270°, and 315° and |**r**| = 1.0 with 0°, 45°, 90°, and 135°.
(PDF)

S8 Fig Perturbed TNs with translocated point charge. |**r**| = 1.0 with 180°, 225°, 270°, and 315° and |**r**| = 2.0 with 0° and 45°.
(PDF)

S9 Fig Perturbed TNs with translocated point charge. |**r**| = 2.0 with 90°, 135°, 180°, 225°, 270°, and 315°.
(PDF)

S1 Table Node label comparison. (PDF)

S2 Table TN parameters. (PDF)
