# Supplementary material for: Prediction of perturbed proton transfer networks
Source: PLoS One. 2018 Dec 12;13(12):e0207718. doi: 10.1371/journal.pone.0207718 (PMC6291078; doi:10.1371/journal.pone.0207718)
Supplement: S2 Fig — q = 0.051 to q = 0.055. (PDF) [file pone.0207718.s002.pdf]

Charge Increase

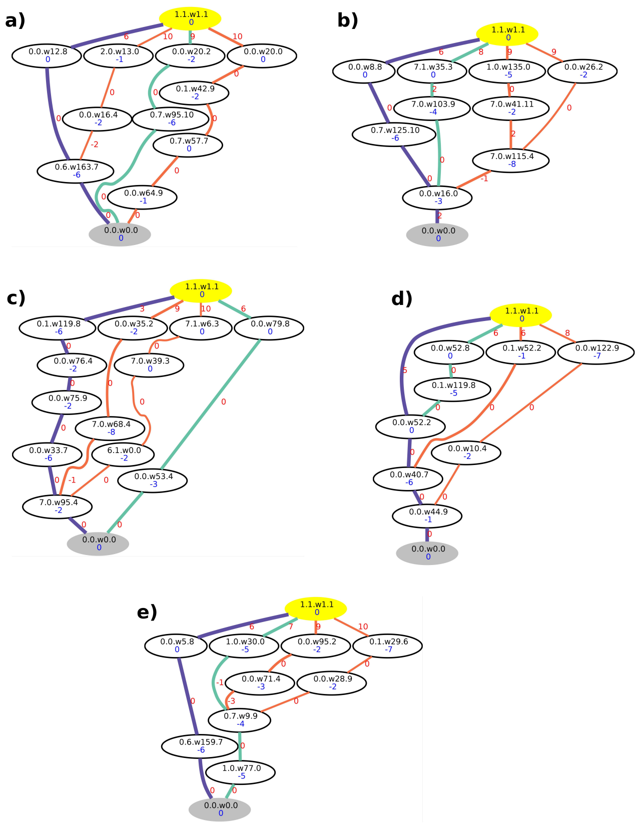

Figure S2: **Perturbed TNs with increased point charge.** Perturbed TNs from a complete network calculation a) $q = 0.051$, b) $q = 0.052$, c) $q = 0.053$, d) $q = 0.054$, and e) $q = 0.055$. The nodes are shown as ellipses, labelled according to the side chain dihedral angles of the carboxylated t-butyl structures, the water pattern and the protonation state (SC0.SC1.Wi.P). The reactant state is shown in grey, the product state in yellow. Edges are shown as lines. Blue numbers represent the energy of the nodes, red numbers the energy of the maximal transition state along the edge used as edge weight. All energies are in kcal/mol, relative to the overall reactant energy and rounded to integer values. The minimax best path is highlighted in indigo, the next best paths are shown in turquoise and orange.
