# Supplementary material for: Prediction of perturbed proton transfer networks
Source: PLoS One. 2018 Dec 12;13(12):e0207718. doi: 10.1371/journal.pone.0207718 (PMC6291078; doi:10.1371/journal.pone.0207718)
Supplement: S1 Table — (PDF) [file pone.0207718.s010.pdf]

Combined Transition Network (TN) from Reidelbach et al [1]

Fig 2 is based on the combined TN from *Reidelbach et al* [1]. The original node labels are provided in Table S1.

Table S1: **Node label comparison.** Corresponding node labels for the TNs from Fig. 2 and the combined TN from *Reidelbach et al* [1].

Nodes from Fig 2	Nodes from *Reidelbach et al* [1]
R	0.0.w0.0
P	0.0.w1.1
a	1.1.w48.7
b	1.0.w27.12
c	0.0.w37.5
d	1.0.w21.11
e	0.1.w43.4
f	1.0.w30.11
g	0.0.w49.11
h	1.0.w20.3
i	0.1.w40.8
j	0.1.w39.8
k	0.2.w231.3
l	0.0.w47.2
m	0.0.w17.3
n	1.0.w46.10
o	1.0.w24.3
p	0.0.w6.14
q	0.0.w4.0
r	0.2.w95.7
s	0.2.w47.3
t,u,v	not present
