# Supplementary material for: Prediction of perturbed proton transfer networks
Source: PLoS One. 2018 Dec 12;13(12):e0207718. doi: 10.1371/journal.pone.0207718 (PMC6291078; doi:10.1371/journal.pone.0207718)
Supplement: S2 Table — (PDF) [file pone.0207718.s011.pdf]

Complete TN Calculations

We performed 51 complete TN calculations. These were the initial TN, 26 TNs with an increased or decreased value of the additional pointcharge and 24 TNs with charge translocations around the initial position. Reactant and product state for the initial TN calculation ($q = 0.050$, $|\mathbf{r}| = 0.0$ Å, $\phi = 0$ °) were derived by re-minimizing the respective states from *Reidelbach et al* [1]. Reactant and product states of all other TN calculation were derived by re-minimizing the respective states from the initial TN calculation. In Table S2 the parameters of all TNs are listed, while Fig S1 to Fig S6 depict the four best proton transfer pathways of each TN.

Table S2: **TN parameters.** Number of nodes and edges per TN, maximal transition barrier and length of the MBP per TN, and added and subtracted nodes and edges per TN with respect to the initial TN for 51 complete TN calculations.

System	Nodes	Edges	ω^* in kcal/mol	Path Length	Node add/sub	Edge add/sub
**Initial**	**252**	**20316**	**5**	**5**	/	/
q	**Charge Increase**					
0.000	276	25918	9	5	175 / 151	21376 / 15774
0.010	264	23688	7	5	155 / 143	18450 / 15078
0.020	273	23507	4	6	148 / 127	17689 / 14498
0.030	260	23255	6	5	127 / 119	16204 / 13265
0.040	268	23165	3	5	136 / 120	16125 / 13276
0.051	259	21572	6	4	110 / 103	13060 / 11804
0.052	232	18747	6	5	89 / 109	10581 / 12150
0.053	243	19583	3	7	93 / 102	10987 / 11720
0.054	240	20189	5	5	98 / 110	12172 / 12299
0.055	250	21101	6	4	102 / 104	12927 / 12142
0.056	251	20237	6	5	116 / 117	13067 / 13146
0.057	258	22129	3	6	110 / 104	14079 / 12266
0.058	252	19716	8	6	116 / 116	12443 / 13043
0.059	243	18720	4	6	109 / 118	11711 / 13307
0.060	239	18380	3	5	99 / 112	11129 / 13065
0.062	246	20499	6	4	109 / 115	12235 / 12052
0.064	234	17755	6	4	102 / 120	10918 / 13479
0.066	246	18964	5	5	107 / 113	11673 / 13025
0.068	235	17607	7	4	101 / 118	10692 / 13401
0.070	235	17527	7	4	108 / 125	11032 / 13821
0.075	229	16650	3	7	104 / 127	10835 / 14501
0.080	232	16816	6	4	111 / 131	11206 / 14706
0.085	240	17522	5	4	128 / 140	12654 / 15448
0.090	226	15674	5	5	114 / 140	10921 / 15563

System	Nodes	Edges	ω^* in kcal/mol	Path Length	Node add/sub	Edge add/sub
0.095	230	15973	3	5	127 / 149	11718 / 16061
0.100	240	17144	3	5	140 / 152	13613 / 16335
$\lvert\mathbf{r}\rvert$, ϕ	**Charge Translocation**					
0.5 Å,0 °	242	18713	9	5	112 / 122	12893 / 14496
0.5 Å,45 °	252	20875	5	6	102 / 102	13145 / 12586
0.5 Å,90 °	241	19426	5	7	73 / 84	9692 / 10582
0.5 Å,135 °	245	20129	8	6	89 / 96	11734 / 11921
0.5 Å,180 °	243	18980	6	4	109 / 118	12746 / 14082
0.5 Å,225 °	257	21059	5	6	98 / 93	12964 / 12221
0.5 Å,270 °	262	22365	6	6	93 / 84	12908 / 10858
0.5 Å,315 °	262	22644	4	4	104 / 95	14383 / 12055
1.0 Å,0 °	250	19817	6	4	139 / 141	15340 / 15839
1.0 Å,45 °	235	17940	5	6	117 / 134	13013 / 15389
1.0 Å,90 °	241	19397	5	4	103 / 114	12393 / 13312
1.0 Å,135 °	257	21073	6	5	114 / 109	14021 / 13264
1.0 Å,180 °	256	22211	6	3	144 / 140	17676 / 15781
1.0 Å,225 °	257	21301	5	6	123 / 118	15213 / 14227
1.0 Å,270 °	267	22542	5	6	122 / 107	15091 / 12865
1.0 Å,315 °	250	20801	6	5	106 / 108	13556 / 13071
2.0 Å,0 °	253	21006	9	4	172 / 171	18356 / 17666
2.0 Å,45 °	248	20770	7	7	149 / 152	17023 / 16569
2.0 Å,90 °	253	21183	5	7	175 / 173	18443 / 17577
2.0 Å,135 °	241	19627	4	4	141 / 152	15667 / 16355
2.0 Å,180 °	270	23634	6	9	191 / 172	20993 / 17674
2.0 Å,225 °	256	21202	5	6	156 / 152	17320 / 16434
2.0 Å,270 °	251	20463	4	5	166 / 167	17352 / 17206
2.0 Å,315 °	238	17961	8	4	138 / 151	14151 / 16507
